# Comparative genomic analysis of eight novel haloalkaliphilic bacteriophages from Lake Elmenteita, Kenya

**DOI:** 10.1371/journal.pone.0212102

**Published:** 2019-02-14

**Authors:** Juliah Khayeli Akhwale, Manfred Rohde, Christine Rohde, Boyke Bunk, Cathrin Spröer, Hans-Peter Klenk, Hamadi Iddi Boga, Johannes Wittmann

**Affiliations:** 1 Leibniz Institute DSMZ–German Collection of Microorganisms and Cell Cultures, Inhoffenstrasse 7B, Braunschweig, Germany; 2 Department of Zoology, Jomo Kenyatta University of Agriculture and Technology, Nairobi, Kenya; 3 Helmholtz Centre for Infection Research, Central Facility for Microscopy, Inhoffenstrasse 7B, Braunschweig, Germany; 4 School of Natural and Environmental Sciences, Newcastle University, Newcastle upon Tyne, United Kingdom; 5 Taita Taveta University College, Voi, Kenya; Fundacao Oswaldo Cruz, BRAZIL

## Abstract

We report complete genome sequences of eight bacteriophages isolated from Haloalkaline Lake Elmenteita found on the floor of Kenyan Rift Valley. The bacteriophages were sequenced, annotated and a comparative genomic analysis using various Bioinformatics tools carried out to determine relatedness of the bacteriophages to each other, and to those in public databases. Basic genome properties like genome size, percentage coding density, number of open reading frames, percentage GC content and gene organizations revealed the bacteriophages had no relationship to each other. Comparison to other nucleotide sequences in GenBank database showed no significant similarities hence novel. At the amino acid level, phages of our study revealed mosaicism to genes with conserved domains to already described phages. Phylogenetic analyses of large terminase gene responsible for DNA packaging and DNA polymerase gene for replication further showed diversity among the bacteriophages. Our results give insight into diversity of bacteriophages in Lake Elmenteita and provide information on their evolution. By providing primary sequence information, this study not only provides novel sequences for biotechnological exploitation, but also sets stage for future studies aimed at better understanding of virus diversity and genomes from haloalkaline lakes in the Rift Valley.

## Introduction

Natural bacteriophage (commonly known as phages) communities are reservoirs of considerable uncharacterized genetic diversity on Earth [1]. Phages are relatively simple in genetic organization and have smaller genomes compared to bacteria [2]. They are tremendously diversified with genome sizes from as low as 17 Kbp up to 0.5 Mbp with high frequency of novel genes found in newly characterized phage genomes [3]. Phage taxonomy has classically depended on the definitions outlined by the International Committee on Taxonomy of Viruses-ICTV [4] which grouped phages based on morphological and behavioral phenotypes. All these fields of analysis merit attention and are actively pursued. However, these approaches lacked direct connection to phage genome sequence which is most credible in tackling phage diversity and provides information necessary to classify phages into groups that reflect their biology [5]. That is why the ICTV intensified its work on the classification of phages using genomic and proteomic approaches [6].

Phage genomics has advanced the use of phages for development of genetic, biotechnological, clinical tools and a large variety of approaches and utilities [7]. Complete phage genomes help to identify conserved sequences referred to as 'signature genes' [8] that facilitate studies of phage evolutionary history and relationships, biodiversity, biogeography and identification of novel phage taxa [5] [9]

The central prerequisite for genome mining as an approach for new natural product discovery is availability of complete genome sequences or genomic sequence data [10]. Genes encoding products likely to be involved in natural products biosynthesis like enzymes can be readily located in sequenced genomes by use of computational sequence comparison tools. The products can then be produced by combinatorial biosynthesis [11]. To gain more insight in molecular biology and characteristics of phages, information on structure, information content and variability of different phage genomes is required. Newly acquired phage DNA therefore provides a reservoir of genetic information for potential use.

In this study, we report complete genome sequences of eight haloalkaliphilic phages; vB_EauM-23, vB_BpsS-36, vB_BpsM-61, vB_BboS-125, vB_EalM-132, vB_BcoS-136, vB_EalM-137 and vB_BpsS-140 isolated from the haloalkaline Lake Elmenteita. The genomes were sequenced, annotated and a comparative and functional analysis using various Bioinformatics tools performed to analyze the sequence data and assess the relatedness of the phages to each other and to those whose sequences are in non-redundant public databases. This study will expand scientific understanding of phage biology and genomic information from the Lake and also significantly add to our understanding of phage diversity in the Lake. This report not only provides novel protein sequences, but also sets the stage for future studies aimed at better understanding virus/host relationships from the haloalkaline lakes in the Rift Valley.

## Materials and methods

Research authorization in Kenya was given by the National Commission for Science, Technology and Innovation (NACOSTI), Kenya Wildlife Service (KWS) and National Environmental Management Authority (NEMA).

### Study site

The study site, Lake Elmenteita, is situated at 0^o^ 27′ S 36^o^ 15′ E on the floor of the Kenyan Rift Valley at 1776 m above sea level and has no direct outlet [12]. The region is characterized by a hot, dry and semi-arid climate with a mean annual rainfall of about 700 mm [13]. Due to the high temperatures there are very high evaporation rates during the drier seasons, leading to a seasonal reduction in the total surface area. The size of Lake Elmenteita is roughly 20 km^2^ and the depth rarely exceeds 1.0 m [14]. The alkalinity of the water is high with a high concentration of carbonates (1200 mg Na_2_CO_3_ l^-1^), chlorides and sulphates [15]. The water temperature ranges between 30 and 40°C and the pH is above 9.

Sediment sample plus the overlying water were collected (March, 2013) into sterile jars, capped on site and preserved in cooled boxes for transportation to the molecular laboratory in Jomo Kenyatta University of Agriculture and Technology (JKUAT). In the laboratory the samples were packaged for transfer to Leibniz Institute—DSMZ (German Collection of Microorganisms and Cell Cultures) in Braunschweig, Germany and stored at 8°C.

Isolation and characterization of bacterial host strains and the corresponding bacteriophages have been described in Akhwale et al (under review, PONE-D-18-23776).

### DNA extraction

DNA was extracted from CsCl purified high-titre stocks of phage using phage DNA isolation kit (Norgen Biotek Corp., Thorold, ON, Canada) according to the manufacturer’s instructions. The purity and the concentration of the DNA were determined using spectrophotometer (Invitrogen Qubit).

### PacBio library preparation and sequencing

Eight haloalkaliphilic phages; vB_EauM-23, vB_BpsS-36, vB_BpsM-61, vB_BboS-125, vB_EalM-132, vB_BcoS-136, vB_EalM-137 and vB_BpsS-140 isolated from the haloalkaline Lake Elmenteita found on the floor of East African Rift Valley in Kenya [16] were sequenced. SMRTbell™ template libraries were prepared according to the instructions from Pacific Biosciences, Menlo Park, CA, USA, following the Procedure and Checklist Greater than 10 kb Template Preparation and Sequencing using a multiplex workflow with symmetric barcoded adapter of 16 nucleotides (F1 to F3), each for one of the phages. Briefly, for preparation of 10kb libraries ~ 4μg genomic DNA isolated from up to eight phages were sheared applying g-tubes™ from Covaris® (Woburn, MA) according to the manufacturer´s instructions. DNAs were end-repaired and ligated overnight to hairpin adapters applying components from the DNA/Polymerase Binding Kit P5 from Pacific Biosciences, Menlo Park, CA, USA, respectively. Reactions were carried out according to the manufacturer´s instructions. DNAs from eight phages were combined equimolar. SMRTbell™ template was exonuclease treated for removal of incompletely formed reaction products. Conditions for annealing of sequencing primers and binding of polymerase to purified SMRTbell™ template were assessed with the Calculator in RS Remote, Pacific Biosciences, Menlo Park, CA, USA. SMRT sequencing was carried out on the PacBio *RSII* (Pacific Biosciences, Menlo Park, CA, USA) taking one 180-minutes movie.

### Demultiplexing, genome assembly and annotation

Data from one SMRT Cell was demultiplexed according to barcodes F1 to F3 using the “RS_Subreads.1” protocol included in SMRTPortal version 2.2.0. Hereby, the “barcoding” option was activated and “symmetric” barcoding was selected in the barcode option pulldown menu. A FASTA-file containing all barcodes was uploaded prior analysis to the “Reference” section of SMRTPortal and selected within the protocol. Output of demultiplexing workflow (barcoded-fastqs.tgz) was used to create whitelists of polymerase reads for each barcode (compare https://github.com/PacificBiosciences/Bioinformatics-Training/wiki/HGAP-Whitelisting-Tutorial). Hereby, a bash script named “Barcode_HGAP.sh“assisted in creating the necessary folder structure, generating the whitelist.txt files as well as the settings.xml file for each subsequent genome assembly. Whitelisted SMRT sequencing data from each phage was assembled independently using the “RS_HGAP_Assembly.3”protocol in SMRTPipe with minimum subread lengths of 1 kb and an estimated genome size of 50 kb. Each phage assembly revealed the fully resolved chromosomes as one contig. The assemblies where either linearized due to recognition of distinct start and end points in the phage assemblies or circularized removing artificial redundancies at the ends of the contigs. Validity of the assemblies was checked using SMRTView and IGV [17]. Finally, the genomes were annotated using Prokka 1.8 [18] with subsequent manual curation in Artemis [19].

### Bioinformatics analyses of sequence data

Two criteria were used to define potential protein coding genes; they had to contain greater than 25 codons and employ ATG, GTG or TTG as initiation codons. Genome size, G+C % content, coding density, total number of genes and additional elements such as inspection of the sequence to search start and termination codons was determined using ARTEMIS tool for sequence visualization [20]. The intergenic genome regions of the phage were searched for transcriptional regulation elements. A search for tRNA genes was done with the tRNAscan-SE program v1.2.1 [21] and ARAGORN v1.2.36 [22].

### Phylogenomic analysis

Dotplots were generated using the DOTMATCHER tool from EMBOSS (Ian Longden, Sanger Institute, Cambridge, UK). VICTOR tool [23] was used to compare phage genomes to other Bacillus viruses. Gene function was predicted by comparing phage ORF sequences against the GenBank nr/nt sequence database using the BLASTp and BLASTn [24] search algorithms and were accepted if the statistical significance of the sequence similarities (E value) was less than 1x10^-5^, the percentage query cover was ≥60% and the percentage identity between the aligned sequences was ≥50%. Predicted amino acid sequences for phage terminase large subunit were used to conduct a phylogenetic analysis of the phages. The eight amino acid sequences were aligned with other phage sequences with known DNA packaging strategies from a reduced set used by Fouts et al [25] using the program ClustalW [26]with default parameters in MEGA v.7 (Pairwise alignment: gap opening penalty = 10, gap extension penalty = 0.1. Multiple alignment: gap opening penalty = 10, gap extension penalty = 0.2. Protein weight matrix = Gonnet. Delay divergent cutoff = 30%) [27]. Phylogenetic tree was inferred using the Maximum—Likelihood method [28] based on the Poisson correction model [29] by applying a bootstrap test with 1000 replicates [30].

## Results

The complete nucleotide sequences of the eight double stranded DNA bacteriophages vary in size from 37, 660 bp-160, 590 bp with a coding density range between 86.0–93.5%. Potential open reading frames (ORFs) of the phages range between 64–240, with majority having ATG as start codon. The basic genome properties (genome size, percentage coding density, number of ORFs, percentage GC content, tRNAs and start codons) of the fully sequenced phages are as summarized in Table 1.

**Table 1:** The basic genomic features of the eight genome sequences of this study.

	Phage	Host	Genome size (bp)	Coding %	ORFs	G +C % content	tRNAs	Transcriptional Terminators	Start Codon		
									ATG	GTG	TTG
1	vB_EauM-23	*Exiguobacterium aurantiacum*	37, 660	91.7	66	52.1	-	6	62	2	2
2	vB_BpsS-36	*Bacillus pseudalcaliphilus*	50, 485	91.6	68	41.1	-	6	62	5	1
3	vB_BpsM-61	*Bacillus pseudofirmus*	48, 160	93.0	75	43.5	-	8	64	11	-
4	vB_BboS-125	*Bacillus bogoriensis*	58, 528	92.2	81	48.6	-	6	81	-	-
5	vB_EalM-132	*Exiguobacterium alkaliphilum*	145, 844	86.0	192	40.6	2	55	181	10	1
6	vB_BcoS-136	*Bacillus cohnii*	160, 590	88.5	240	32.2	17	15	202	17	21
7	vB_EalM-137	*Exiguobacterium alkaliphilum*	41, 601	91.2	64	50.9	-	8	60	2	2
8	vB_BpsS-140	*Bacillus pseudalcaliphilus*	55, 091	91.0	68	39.8	-	4	64	2	2

Two tRNA genes (Asn_gtt_ and Arg_tct_) were detected in the genome of vB_EalM-132, and 17 tRNAs were found clustered in the DNA replication and metabolism region (bp 54444 to 56828) in vB_BcoS-136 (S1 Table). The rest of the phage genomes in this study had no tRNAs.

**Fig 1:**
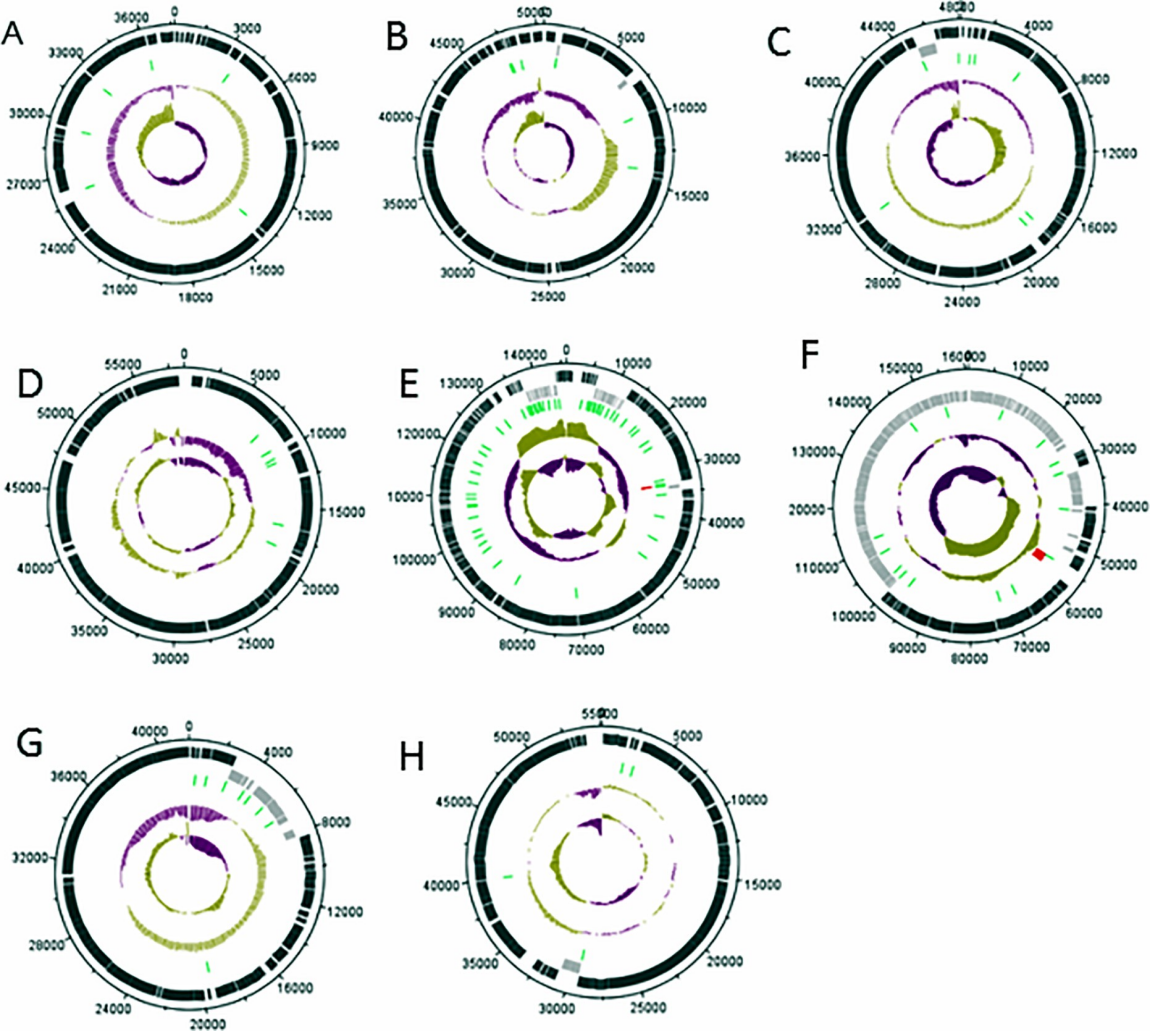
Genome maps drawn to scale, displaying regions and features of interest. A (vB_EauM-23), B (vB_BpsS-36), C (vB_BpsM-61), D (vB_BboS-125), E (vB_EalM-132), F (vB_BcoS-136), G (vB_EalM-137) and H (vB_BpsS-140). First track (black) show forward transcribed ORFs and second track (grey) show reverse transcribed ORFs respectively. Third track (green) show terminators and fourth track (red) show tRNAs. Moving inward, the track show the %GC content (purple = low %GC) and innermost of the genome map GC skew ([G-C]/[G+C]).

Genome organization as indicated by genetic maps show ORFs distributed on both forward and reverse strands, apart from phages vB_EauM-23 and vB_BboS-125 that have all ORFs located on the forward strand as shown in Fig 1.

Genome wide comparisons with sequences in the GenBank nr/nt database revealed no significant matches. Phage vB_EalM-132 shares very low similarity with well-studied Bacillus phage SP01 (GenBank: KC595513.2), a representative phage of the *Myoviridae* family [31] and Bacillus phage CP-51 (GenBank: KF554508.2) [32]. Phage vB_BpsS-136 also had very low similarity to Bacillus phage vB_BanS-Tsamsa (GenBank: KC481682.1), barely detectable by a diagonal dotplot analysis. The graphical representation of the regions of similarity can be seen in dotplot analyses of the genomes generated using the DOTMATCHER tool (S1 Fig).

**Fig 2:**
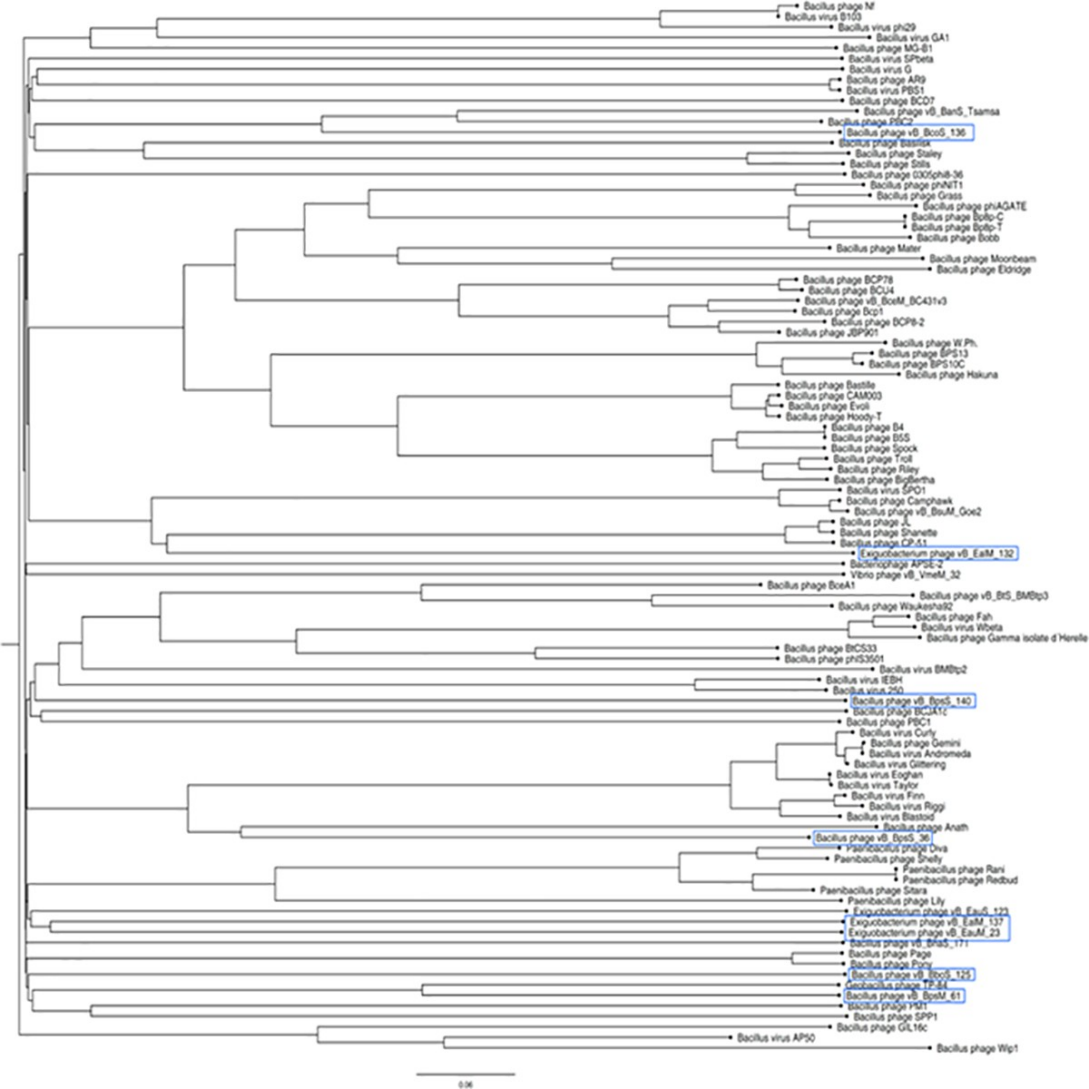
Phylogenomic Genome BLAST Distance Phylogeny (GBDP) tree of isolated phages of this study compared to Bacillus phages. Tree was generated by VICTOR and visualized with Fig Tree. Phages of this study are marked in blue.

Phylogenomic analysis of the phage genomes compared to other *Bacillus* viruses using the VICTOR tool at the nucleotide level showed the phages of this study were not grouped into known clusters and formed distinct branches of their own as shown in Fig 2.

**Fig 3:**
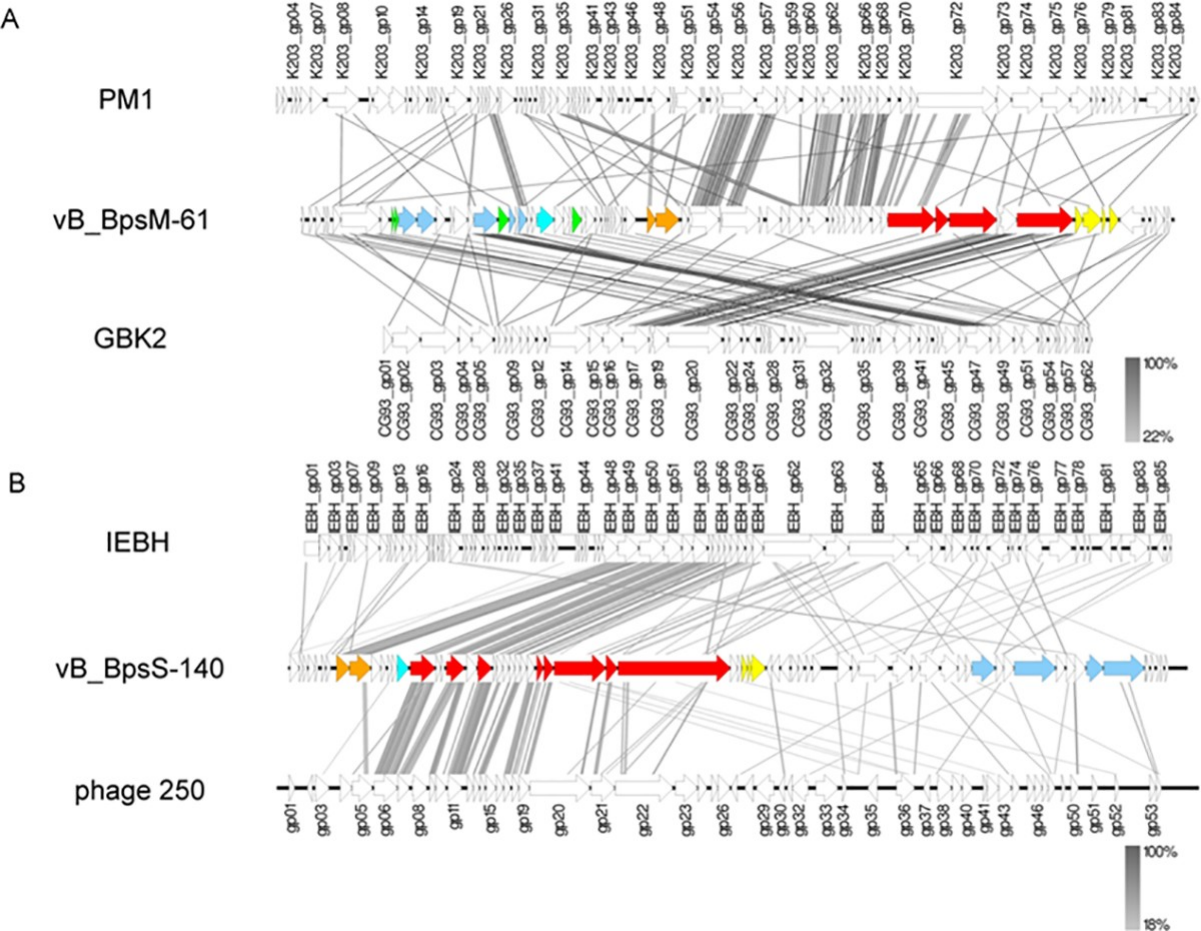
**Genomic comparisons of vB_BpsM-61 (A) and vB_BpsS-140 (B) with related phages.** Functional clusters are marked by the same color, namely DNA packaging (orange), structural components (red), lysis (yellow), regulation (green), replication (blue) and nucleotide metabolism (turquois). Synteny plot was generated with Easyfig with amino acid sequence comparison [33]. The amino acid sequence identity range is indicated by a gradient scale.

At the amino acid level phages of our study revealed homologies to already described phages. vB_BpsM-61 showed weak similarities to *Bacillus* phage PM1 and *Geobacillus* phage GBK2 in parts of its cluster for structural components, while vB_BpsS-140 encoded genes for proteins similar to homologs of a terminase and several structural proteins of *Bacillus* phage IEBH and Bacillus phage 250, respectively (Fig 3A and 3B).

**Fig 4:**
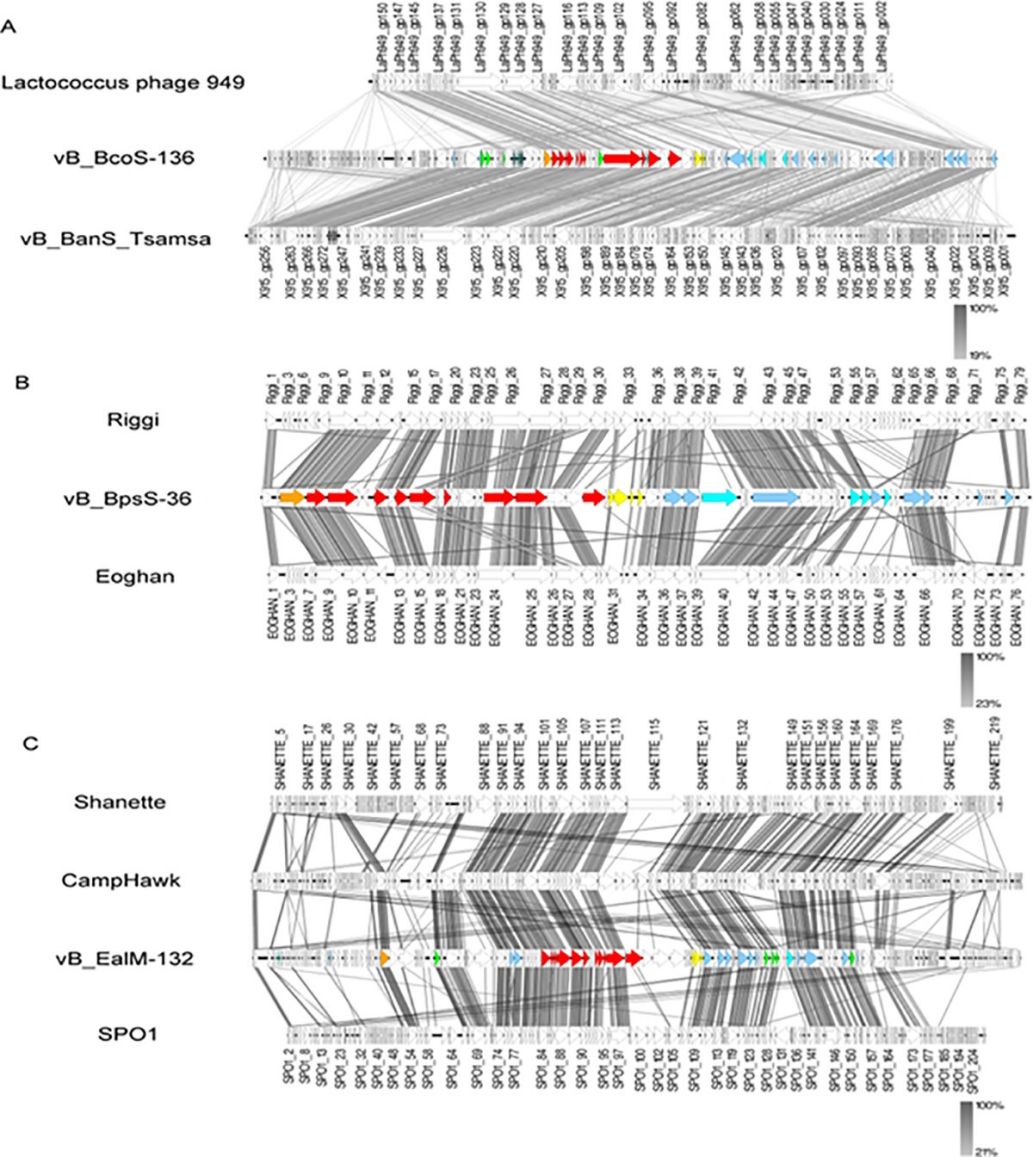
**Genomic comparisons of vB_BcoS-136 (A), vB_BpsS-36 (B) and vB_EalM-132 (C) with related phages.** Functional clusters are marked by the same color, namely DNA packaging (orange), structural components (red), lysis (yellow), regulation (green), replication (blue) and nucleotide metabolism (turquois). Synteny plot was generated with Easyfig with amino acid sequence comparison [33]. The amino acid sequence identity range is indicated by a gradient scale.

While comparison to known phages resulted in the detection of homologies to only short areas with weak similarities, the genomes of vB_BcoS-136, vB_BpsS-36 and vB_EalM-132 showed homologous regions to *Bacillus* phages Tsamsa (Fig 4A), Riggi (Fig 4B) and CampHawk (Fig 4C), respectively, over wider ranges.

**Fig 5:**
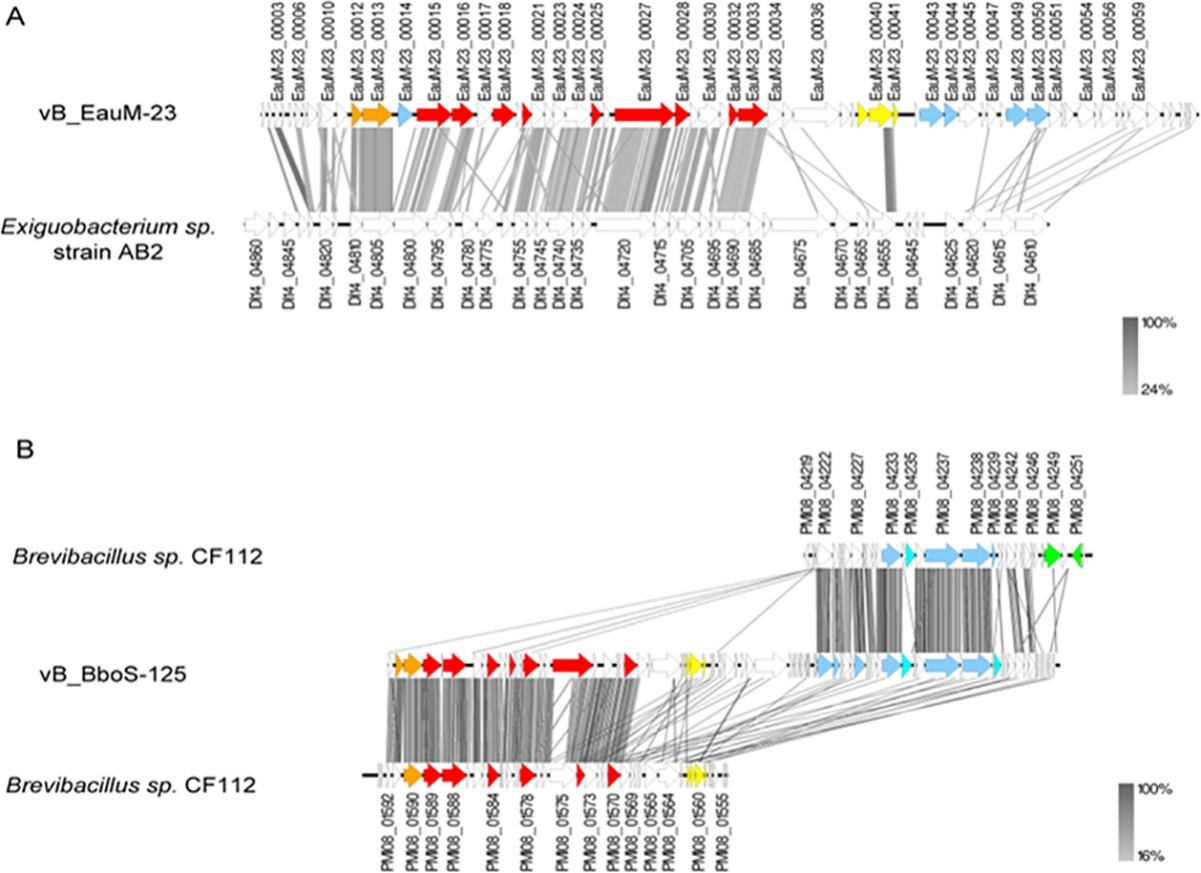
**Genomic comparisons of isolated phages vB_EauM-23 (A) and vB_BboS-125 (B) of this study with not annotated prophage regions in bacterial genomes.** Functional clusters are marked by the same colour, namely DNA packaging (orange), structural components (red), lysis (yellow), regulation (green), replication (blue) and nucleotide metabolism (turquois). Synteny plot was generated with Easyfig with amino acid sequence comparison [33]. The amino acid sequence identity range is indicated by a gradient scale.

When compared to GenBank database, only weak similarities to other phages were detected for phages vB_EauM-23 and vB_BboS-125. Instead, in particular deduced amino acid sequences of genes of the clusters for structural components and DNA packaging showed homologies to genomic regions in *Exiguobacterium* sp. strain AB2 and *Brevibacillus* sp. strain CF112, respectively, which we assume to be not-annotated prophage regions. Additionally, for vB_BboS-125, similarities to genes with conserved domains of the replication cluster, e.g. a helicase and primase, were found in a different part of the draft genome of *Brevibacillus* sp. strain CF112 (Fig 5A and 5B).

To get insights into possible mechanisms of DNA packaging, the amino acid sequence of conserved structural protein; large terminase subunit (terL) was chosen for phylogenetic analysis.

**Fig 6:**
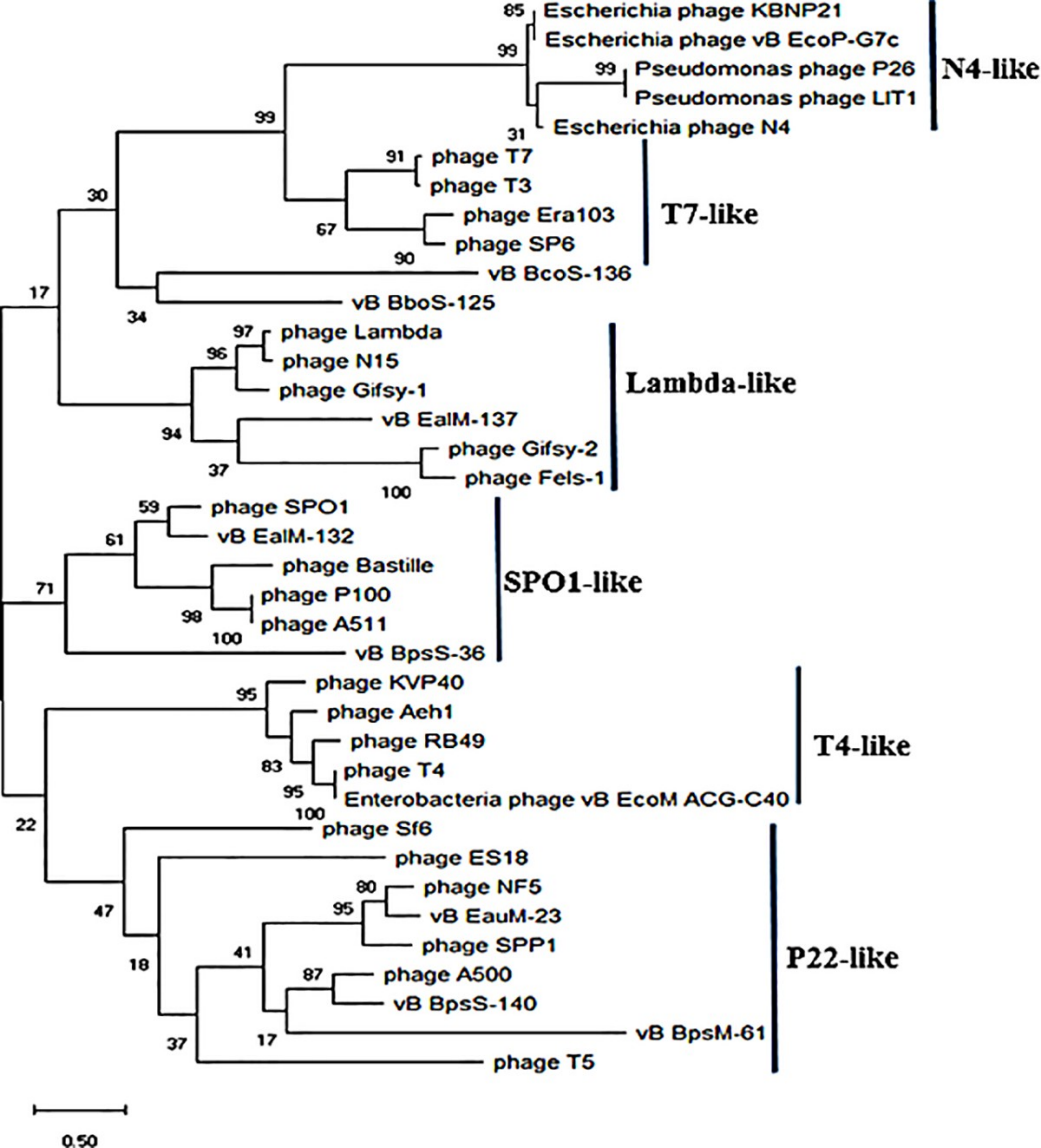
Phylogenetic analysis of large terminase subunits compared to phages with known DNA packaging strategies. The maximum—Likelihood tree was inferred based on ClustalW alignment of large terminase subunits amino acid sequences. The tree was rooted via midpoint rooting [34]. The numbers at the nodes represent bootstrap values based on 1,000 resamplings.

Phylogenetic analysis of large terminase subunits with other phages of known DNA packaging strategies from a reduced set used by Fouts et al [25] using a Maximum—Likelihood method revealed that phage vB_EalM-137 cluster together with Lambda-like phages with a bootstrap value of 94%, vB_BpsS-36 and vB_EalM-132 cluster together with SP01-like phages with a bootstrap value of 71%, vB_EauM-23, vB_BpsM-61 and vB_BpsS-140 cluster together with P22-like phages with a bootstrap value of 47%. Phages vB_BboS-125 and vB_BcoS-136 terminase differ from previously described phages as they did not cluster with any of the phages with already known DNA packaging strategy, but formed a distinct phyletic line (Fig 6).

Analysis using BLASTP show that all phages of this study contain amidase endolysin belonging to a class of N-acetylmuramyl-L-Ala-amidases. Only phage vB_BpsS-140 had endopeptidase endolysin. Summaries on phage-derived endolysins are found in Table 2

**Table 2:** Summary of enzymatic activity of phage-derived endolysins of the phages of this study.

	Phage	Gene	Position	Putative enzymatic activity	Best blast_p_ hit	% identity	E-Value	aa length
1	vB_EauM-23	cw1C	24398–25387	Amidase	N-acetylmuramoyl-L-alanine amidase [*Oceanobacillus iheyensis*] Sequence ID: WP_011064621.1	46	1e-47	329
2	vB_BpsS-36	lytC	23151–24131	Amidase	N-acetylmuramoyl-L-alanine amidase [*Bacillus megaterium*] Sequence ID: WP_098325513.1	51	8e-56	326
3	vB_BpsM-61	xlyA	43132–44160	Amidase	N-acetylmuramoyl-L-alanine amidase [*Bacillus halosaccharovorans*] Sequence ID: WP_078433380.1	43	9e-73	342
4	vB_BboS-125	xlyA	8087–9184	Amidase	N-acetylmuramoyl-L-alanine amidase [*Clostridium* sp. Bc-iso-3] Sequence ID: WP_069195874.1	50	7e-40	365
5	vB_EalM-132	xlyA_1	83894–85006	Amidase	N-acetylmuramoyl-L-alanine amidase [*Bacillus assilioanorexius*] Sequence ID: WP_019243634.1	70	2e-84	370
6	vB_BcoS-136	cw1C	94498–95766	Amidase	N-acetylmuramoyl-L-alanine amidase [*Thermoactinomyces* sp. DSM 45892].	68	3e-78	422
7	vB_EalM-137	cw1C	1243–2262	Amidase	N-acetylmuramoyl-L-alanine amidase [*Oceanobacillus iheyensis*] Sequence ID: WP_011067538.1	44	4e-41	339
8	vB_BpsS-140	cw1K	28361–29254	Endopeptidase	peptidoglycan L-alanyl-D-glutamate endopeptidase CwlK [*Terribacillus aidingensis*] Sequence ID: SNZ14541.1	54	4e-112	297

A wide assortment of conserved replication factors were detected in the genomes. Phages vB_BpsS-140, vB_BcoS-136 and vB_BpsS-36 use DNA polymerase lll subunit alpha enzyme for DNA replication. Phages vB_BboS-125 and vB_EalM-132 employ DNA polymerase l for replication. Phages vB_EalM-137, vB_BpsM-61 and vB_EauM-23 did not encode the polymerase enzyme (Table 3).

**Table 3:** Replication factor DNA polymerase in the eight phages.

	Phage	ORF no.	DNA polymerase gene	Bases	GC%
1	vB_EauM-23		-		
2	vB_BpsS-36	39	DNA polymerase lll subunit alpha (dnaE)	3087	41.6
3	vB_BpsM-61		-		
4	vB_BboS-125	48	DNA polymerase l (pol A)	2553	49.2
5	vB_EalM-132	123	DNA polymerase l (pol A_1)	2751	39.7
6	vB_BcoS-136	166	DNA polymerase lll subunit alpha (dnaE)	3306	33.3
7	vB_EalM-137		-		
8	vB_BpsS-140	61	DNA polymerase lll subunit alpha (dnaE_1)	1065	41.9
		62	DNA polymerase lll subunit alpha (dnaE_2)	2520	40.8
		49	DNA polymerase lll polC—type (polC)	777	40.2

Further analysis of these genomes revealed few more genes for proteins with conserved domains. Gene 00143 of phage vB_BcoS-136 (position 73375–74382) and 00008 of phage vB_EalM-137 (position 4408–5544) encode integrase enzyme. Phage vB_EauM-23 has a putative dUTPase (_00010), putative HNH endonuclease (_00014), two proteins for replication containing a DnaD (_00049) and DnaC domain (_00050) respectively. Phage vB_BpsS-36 has two proteins for replication containing a DnaB (_00031) and DnaG (_00032) domain respectively, putative DNA translocase FtsK (_00053), and an endonuclease (_00066). Phage vB_BpsM-61 has a replicative DNA protein DnaC (_00017), helix destabilizing Ssb protein (_00021), a putative dUTPase (_00025), a putative Holliday junction resolvase (_00027). Phage vB_EalM-132 has a replicative DNA protein DnaC (_00100), putative exonuclease (_00129), an ATP-dependent DNA helicase (_00141) and NAD-dependent protein acetylase (_00142). Phage vB_BcoS-136 has a gene for peptidase (_00036), putative DNA ligase (00136), DNA gyrase subunit A (_00210) and B (00211) respectively, and ribonuclease HI (_00235). Phage vB_EalM-137 has a gene for helix destabilizing Ssb protein (_00021), a putative Holliday junction resolvase (_00026), a putative dUTPase (_00029). Phage vB_BpsS-140 has a putative recombinase (_00051), a putative DNA primase (_00057), a putative adenylate kinase (_00058).

## Discussion

Obtaining the complete genome sequence of phage is an essential prerequisite for any type of functional genomics and phage-based applications. Genome sequences of phage also provide many insights on the biology and ecology of phage. The basic genome properties of the phages of this study including genome size, percentage coding density, number of ORFs, percentage GC content, tRNAs and gene organizations reveal no relationship of the phages to each other.

Only phages vB_EalM-132 and vB_BcoS-136 encoded two and seventeen tRNA genes respectively. The rest of the phages of this study did not have the translation associated gene, hence assumption that the phages are well adapted to their hosts in regard to codon usage and do not require additional tRNAs of their own for regulation of transcription [35].

Genome wide comparisons with sequences in the GenBank database revealed the genomes of our phages had no significant matches hence novel. This was supported by the phylogenomic analysis at the nucleotide level using the VICTOR tool. This shows the phage gene pool in the Haloalkaline Lake is still largely unexplored. While no or only weak similarities were detected at the nucleotide level, at the amino acid level phages of our study revealed mosaicism [36] to already described phage structural, functional, lysis, regulation, replication and nucleotide metabolism genes. This indicates extensive horizontal gene transfer among the genomes of this study and phages of other bacterial species.

The large terminase subunit is considered the most universally conserved gene sequence in phages [37] hence used to infer phylogeny to decipher evolutionary relationships among phages belonging to different families [38]. Comparing amino acid sequence for each predicted terminase large subunit protein with homologous terminase sequences of well-characterized phage terminase, provides insight into predicting DNA packaging strategy of uncharacterized phages. Phage vB_EalM-137 cluster together Lambda-like phages with cohesive ends [39]. vB_EauM-23, vB_BpsM-61 and vB_BpsS-140 cluster together with P22 which is the best characterized of phages that show headful packaging strategy [40]. vB_BpsS-36 and vB_EalM-132 cluster together with the best studied *Bacillus subtilis* phage SP01 that show long exact direct repeat ends [41]. However, Phage vB_BboS-125 and vB_BcoS-136 terminase did not cluster with previously described phages but formed a distinct phyletic line, which suggest that they maybe a new genus of bacteriophages.

Endolysins are produced during the late stages of phage infection and are designed to attack the peptidoglycan that holds the bacterial cell together to release phage progeny [42]. Endolysins of tailed phages are capable of killing susceptible organisms when applied exogenously as recombinant proteins [43], therefore have a high potential for application in therapy and disease control, because of their diversity and specificity [44]. Phages of this study contain amidase endolysin belonging to a class of N-acetylmuramyl-L-Ala-amidases. Only phage vB_BpsS-140 had endopeptidase endolysin.

Gene 00143 of phage vB_BcoS-136 (position 73375–74382) and 00008 of phage vB_EalM-137 (position 4408–5544) encode integrase enzyme responsible for phage integration into and excision from bacteria chromosome hence temperate phages. The rest of phages of this study did not encode integrase hence proceed through the lytic life cycle. Phage integrases have a growing importance for genetic manipulation of living eukaryotic cells, especially those with large genomes such as mammals and most plants, since they can mediate efficient site-specific recombination between two different sequences, for which there are few tools for precise manipulation of the genome [45][46].

Comparative genomic approaches using closely related phages from different host organisms can therefore fill gap in protein function assignment. Some information can be deduced from the genetic context or location of genes of interest, because phage genomes are organized in a modular fashion and mosaicism i.e. the exchange of genetic modules between phages [47].

## Conclusion

Sequencing, annotation, genome analysis and function prediction was the accomplishment of this study. The bioinformatics analysis of the genomes showed diversity among the phages. The unraveling of each novel phage genome has provided supply of proteins for further exploitation for biological and biotechnological ends. Our research also contributes to the diversity of phage sequences in the DNA database and makes their respective genomes useful as comparisons for future gene annotations. A useful endeavor would be the determination of currently unknown gene functions through study of bacteriophage gene expression. By connecting genes with structure and function, we would be able to better understand phage biology. Recent advances in genome sequencing, comparative genomics combined with functional genomic studies will undoubtedly play a major role in filling this knowledge gap and increase our understanding of phage biology for better utilization. The continued pursuit of phage whole genome sequencing will therefore increase the value of the virome data and offer profuse insights into the diversity of phages in the haloalkaline lake Elmenteita.

## Nucleotide sequence accession numbers

The genome sequences were deposited at NCBI GenBank using Bankit under the following accession numbers: MH844558 (vB_EauM-23), MH884513 (vB_BpsS-36), MH884514 (vB_BpsM-61), MH884509 (vB_BboS-125), MH884511 (vB_EalM-132), MH884508 (vB_BcoS-136), MH884510 (vB_EalM-137) and MH884512 (vB_BpsS-140).

## Supporting information

S1 Table Table showing tRNAs in phages vB_EalM-132 (2) and vB_BcoS-136 (17), their respective positions in the genome, number of bases and %GC content. (DOCX)

S1 Fig Comparative nucleotide sequence analysis of vB_EalM-132 against Bacillus Phage SP01 and Bacillus Phage CP-51 respectively. Local regions of similarity are indicated by the diagonal line. Windows size of 150 and threshold value of 50 were used as parameters for Dotmatcher program.
(TIF)
