# Supplementary material for: Comparative genomic analysis of eight novel haloalkaliphilic bacteriophages from Lake Elmenteita, Kenya
Source: PLoS One. 2019 Feb 14;14(2):e0212102. doi: 10.1371/journal.pone.0212102 (PMC6375668; doi:10.1371/journal.pone.0212102)
Supplement: S1 Table — (DOCX) [file pone.0212102.s001.docx]

|  | | **vB_EalM-132** | | | **vB_BcoS-136** | | |
| --- | --- | --- | --- | --- | --- | --- | --- |
| **No.** | **tRNA** | **Position** | **bases** | **%GC** | **Position** | **bases** | **%GC** |
| 1 | tRNA-Asn(gtt) | 33473,33545 | 73 | 49.3 | 54444,54518 | 75 | 49.3 |
| 2 | tRNA-Asp(gtc) |  |  |  | 54522,54596 | 75 | 49.3 |
| 3 | tRNA-Ala(tgc) |  |  |  | 54841,54912 | 72 | 45.8 |
| 4 | tRNA-Gln(ttg) |  |  |  | 54925,55001 | 77 | 46.8 |
| 5 | tRNA-Trp(cca) |  |  |  | 55006,55079 | 74 | 45.9 |
| 6 | tRNA-His(gtg) |  |  |  | 55246,55316 | 71 | 49.3 |
| 7 | tRNA-Glu(ttc) |  |  |  | 55653,55727 | 75 | 48.0 |
| 8 | tRNA-Ile(gat) |  |  |  | 55729,55803 | 75 | 37.3 |
| 9 | tRNA-Tyr(gta) |  |  |  | 55807,55891 | 85 | 49.4 |
| 10 | tRNA-Leu(tag) |  |  |  | 56065,56139 | 75 | 52.0 |
| 11 | tRNA-Pro(tgg) |  |  |  | 56144,56221 | 78 | 50.0 |
| 12 | tRNA-Thr(tgt) |  |  |  | 56223,56297 | 75 | 42.7 |
| 13 | tRNA-Arg(acg) |  |  |  | 56315,56388 | 74 | 47.3 |
| 14 | tRNA-Ser(tga) |  |  |  | 56488,56581 | 94 | 48.9 |
| 15 | tRNA-Ser(gct) |  |  |  | 56588,56679 | 92 | 58.7 |
| 16 | tRNA-Arg(tct) | 33244,33319 | 76 | 46.1 | 56681,56754 | 74 | 50.0 |
| 17 | tRNA-Gly(tcc) |  |  |  | 56758,56828 | 71 | 53.5 |
