# Supplementary figures and images for: Comparative genomic analysis of eight novel haloalkaliphilic bacteriophages from Lake Elmenteita, Kenya
Source: PLoS One. 2019 Feb 14;14(2):e0212102. doi: 10.1371/journal.pone.0212102 (PMC6375668; doi:10.1371/journal.pone.0212102)

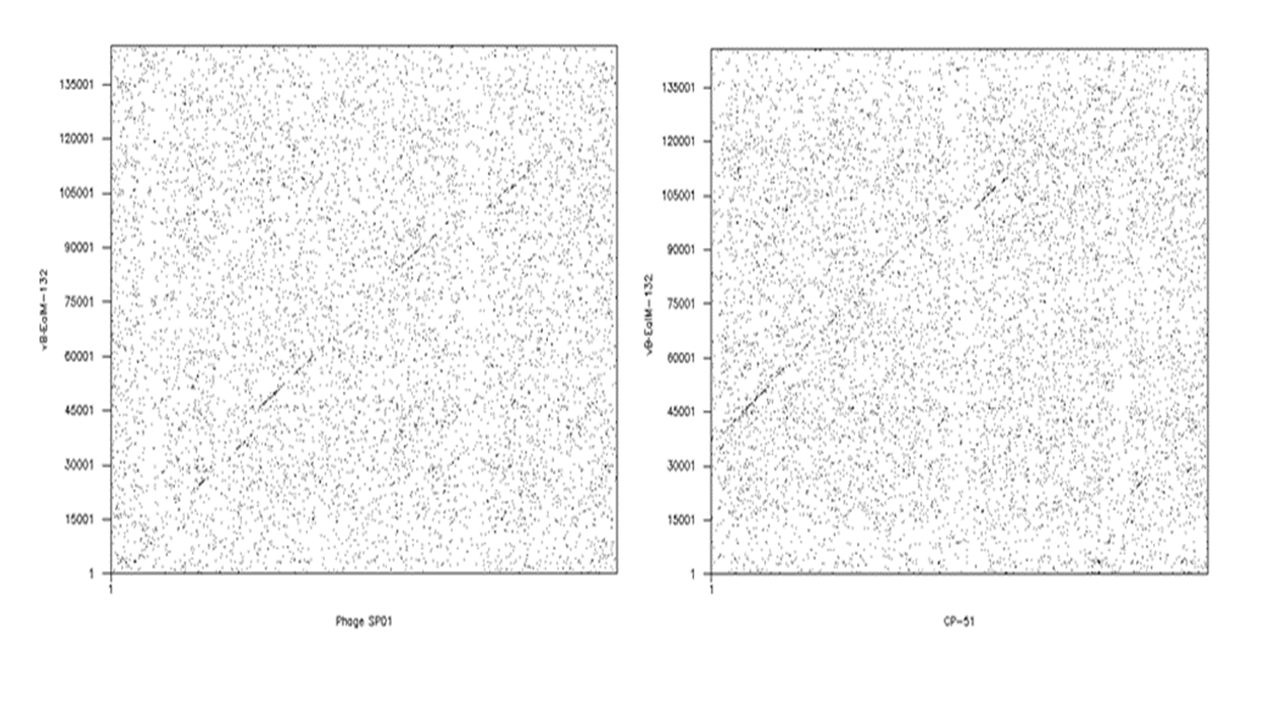

Supplement: S1 Fig — Local regions of similarity are indicated by the diagonal line. Windows size of 150 and threshold value of 50 were used as parameters for Dotmatcher program. (TIF) [file pone.0212102.s002.tif]
